# Use of systemic glucocorticoids and risk of breast cancer in a prospective cohort of postmenopausal women

**DOI:** 10.1186/s12916-021-02004-6

**Published:** 2021-08-02

**Authors:** Manon Cairat, Marie Al Rahmoun, Marc J. Gunter, Pierre-Etienne Heudel, Gianluca Severi, Laure Dossus, Agnès Fournier

**Affiliations:** 1 Nutrition and Metabolism Branch, International Agency for Research on Cancer, Lyon, France; 2 Université Paris-Saclay, UVSQ, Inserm, Gustave Roussy, Exposome and heredity team, CESP, F-94805 Villejuif, France; 3 Medical Oncology Department, Centre Léon Bérard, Lyon, France; 4 Department of Statistics, Computer Science and Applications “G. Parenti” (DISIA), University of Florence, Florence, Italy

**Keywords:** Glucocorticoids, Breast cancer, Molecular status, Cancer stage, Oestrogen receptors, Postmenopausal women, Cohort, Pharmacoepidemiology

## Abstract

**Background:**

Glucocorticoids could theoretically decrease breast cancer risk through their anti-inflammatory effects or increase risk through immunosuppression. However, epidemiological evidence is limited regarding the associations between glucocorticoid use and breast cancer risk.

**Methods:**

We investigated the association between systemic glucocorticoid use and breast cancer incidence in the E3N cohort, which includes 98,995 women with information on various characteristics collected from repeated questionnaires complemented with drug reimbursement data available from 2004. Women with at least two reimbursements of systemic glucocorticoids in any previous 3-month period since January 1, 2004, were defined as exposed. We considered exposure as a time-varying parameter, and we used multivariable Cox regression models to estimate hazard ratios (HRs) of breast cancer. We performed a competing risk analysis using a cause-specific hazard approach to study the heterogeneity by tumour subtype/stage/grade.

**Results:**

Among 62,512 postmenopausal women (median age at inclusion of 63 years old), 2864 developed breast cancer during a median follow-up of 9 years (between years 2004 and 2014). Compared with non-exposure, glucocorticoid exposure was not associated with overall breast cancer risk [HR = 0.94 (0.85–1.05)]; however, it was associated with a higher risk of in situ breast cancer and a lower risk of invasive breast cancer [HR_*insitu*_ = 1.34 (1.01–1.78); HR_invasive_ = 0.86 (0.76–0.97); *P*_*homogeneity*_ = 0.01]. Regarding the risk of invasive breast cancer, glucocorticoid exposure was inversely associated with oestrogen receptor (ER)-positive breast cancer [HR_ER+_ = 0.82 (0.72–0.94); HR_ER−_ = 1.21 (0.88–1.66); *P*_*homogeneity*_ = 0.03]; it was also inversely associated with the risk of stage 1 or stage 2 tumours but positively associated with the risk of stage 3/4 breast cancers [HR_stage1_ = 0.87 (0.75–1.01); HR_stage2_ = 0.67 (0.52–0.86); HR_stage3/4_ = 1.49 (1.02–2.20); *P*_*homogeneity*_ = 0.01].

**Conclusion:**

This study suggests that the association between systemic glucocorticoid use and breast cancer risk may differ by tumour subtype and stage.

**Supplementary Information:**

The online version contains supplementary material available at 10.1186/s12916-021-02004-6.

## Background

Synthetic glucocorticoids, drugs that are structurally and pharmacologically similar to the endogenous hormone cortisol, are used to treat a wide range of diseases, most often chronic diseases such as rheumatologic disorders, autoimmune diseases, allergies, or respiratory diseases [1]. Glucocorticoids possess various anti-inflammatory, immunosuppressive, metabolic, and endocrine properties, which have been hypothesized to be potentially either harmful or beneficial regarding breast cancer development [2, 3]. Indeed, on one hand, it has been suggested that glucocorticoids might prevent breast cancer by decreasing the levels of various mediators such as oestrogens, pro-inflammatory cytokines, and eicosanoids, potentially involved in the pathophysiology of breast cancer [2–4]; oestrogen inhibition would mostly prevent oestrogen receptor-positive (ER+) breast cancer. On the other hand, glucocorticoids might promote breast cancer progression by facilitating tumour cells to escape from immune surveillance [5], promoting metabolic dysfunction [6], or inducing insulin resistance [7, 8], all suspected risk factors for breast cancer development [9–11]. To date, there is a lack of epidemiological studies addressing the relationship between synthetic glucocorticoid use and breast cancer risk. To our knowledge, only two epidemiological studies have been published, both based on data from nationwide medico-administrative databases from Northern Denmark [12, 13]. The most recent study, which was an extension of the previous one [12], reported no association between at least three prescriptions of systemic or inhaled glucocorticoids and risk of invasive breast cancer [13]. There was also no association when the authors categorized glucocorticoid exposure into recent and former use, according to intensity or duration of use, or stratified the analyses by menopausal status. However, they were not able to stratify their analyses by breast cancer subtype, even though the various pharmacological properties of glucocorticoids could influence differently these subtypes. Considering breast cancer subtypes could therefore help understand which mechanisms are at play in the glucocorticoids-breast cancer associations.

To advance knowledge on the role of glucocorticoids on breast cancer development, we evaluated the associations between systemic glucocorticoid use and breast cancer incidence, overall and by breast cancer subtype, in the E3N (Etude Epidémiologique auprès de femmes de la Mutuelle Générale de l’Education Nationale) cohort.

## Methods

### E3N cohort

The E3N study is an ongoing prospective cohort, which initially aimed to investigate the relationship between lifestyle, diet, hormones, environment, and female cancers [14]. In 1990, 499,668 French women born between 1925 and 1950, living in metropolitan France, and insured by a specific national health scheme covering mainly teachers were invited to participate in the study. Among them, 98,995 agreed to participate, signed a written informed consent, and responded to a first questionnaire, which included questions on socio-demographic factors (educational level and current employment status), anthropometric measures (current and at different times in life), menstrual and reproductive factors (age at menarche, parity, age at first pregnancy, breastfeeding, and menopausal status), lifetime medical and surgical history, family history of cancer, gynaecological follow-up (pap smear frequency and date of the last mammogram), lifetime tobacco consumption, and current physical activity. Every 2 or 3 years thereafter, participants completed self-administered follow-up questionnaires to update previous information or to collect new ones. For each cohort member, the health insurance plan provided data on all outpatient reimbursements for health expenditure since January 1, 2004. The E3N cohort was conducted in accordance with the Declaration of Helsinki and received ethical approval from the French National Commission for Data Protection and Privacy.

### Study population and follow-up

Follow-up started on July 1, 2004 (study baseline) and ended at the date of diagnosis of any cancer (except for basal cell carcinoma and in situ colorectal tumour), the latest completed questionnaire, or November 17, 2014 (the date at which the last considered E3N questionnaire was sent to participants), whichever occurred first. The selection of the study population has been already described previously [15]. In brief, we excluded E3N participants (i) with no healthcare reimbursement during the year 2004, (ii) with no follow-up after study baseline, (iii) who did not answer before the study baseline to the questionnaire sent in 2002, and (iv) diagnosed with cancer (except basal cell carcinoma and in situ colorectal tumours) before the study baseline. In addition, few women had not reached menopause at study baseline (*n* = 1529) and were therefore excluded. Thus, our study population included 62,512 E3N postmenopausal women.

### Identification of breast cancer cases

Most breast cancer cases were self-reported in the questionnaires or, to a lesser extent, spontaneously reported by participants’ next of kin or identified from the cause of death data. Among self-reported cases for which we could obtain medical information (from pathology reports, other medical documents, or through contact with participants’ physicians), less than 5% were not confirmed by the medical information obtained, and these false-positive self-reports were considered as non-cases. Due to the low proportion of false-positive self-reports, self-reported cases for which we could not obtain medical information were considered as true cases. Pathology reports, which were obtained for 95% of the incident cases identified in the entire cohort, were used to extract information on tumour characteristics such as invasiveness status (in situ/invasive/unknown), oestrogen receptor (ER) status (ER-positive/ER-negative/unknown), progesterone receptor (PR) status (PR-positive/PR-negative/unknown), human epidermal growth factor receptor 2 (HER2) status (HER2-positive/HER2-negative/unknown), histological subtype (ductal/lobular/others/unknown), grade (from 1 to 3 or unknown), and stage (from 1 to 4 or unknown).

### Exposure to systemic glucocorticoids

We considered all deliveries of systemic glucocorticoids (anatomical therapeutic chemical codes: H02AB01, H02AB03, H02AB04, H02AB06, H02AB07, H02AB08, H02AB09, and H02AB17) since January 1, 2004. The following data were extracted for each glucocorticoid delivery: date of purchase, active ingredient, number of pills/phials per package, and route of administration.

We defined “recurrent” users as women with at least two reimbursements of the drug of interest during any previous 3-month period since January 1, 2004. Other women were considered as never/occasional users and served as the reference category. We classified exposure among recurrent users according to the following characteristics: type of glucocorticoid (active ingredient), route of administration, time since the first or the last use, age at first use, and cumulative number of reimbursements. The date of last use was calculated as the date of last purchase + the number of pills/phials contained in the last reimbursed box. Women with glucocorticoid reimbursements between January 1, 2004, and April 1, 2004, were likely to have begun their treatment before the availability of reimbursement data, and in that case, the cumulative number of reimbursements, age at first use, and time since the first use might be left-truncated. Thus, unless they could be assigned to the highest category of number of reimbursements/time since first use or the lowest category of age at first use, we assigned these women to an “unknown” category.

### Covariates

Variables considered as potential confounders are listed in Table 1. The number of consultations with any doctor during the preceding 6 months and “recurrent” use (defined as at least two reimbursements of the drug of interest during any previous 3-month period since January 1, 2004) of other drugs likely to be also used by glucocorticoid users [paracetamol (anatomical therapeutic chemical code: N02BE01), nonsteroidal anti-inflammatory drugs (M01A), proton-pump inhibitors (A02BC), immunosuppressants (L04), and symptomatic slow-acting drugs for osteoarthritis (glucosamine: M01AX05, diacerein: M01AX21, oxaceprol: M01AX24, chondroitin sulfate: M01AX25, or avocado and soybean oil: M01AX26)] were identified using the drug reimbursement database. Lifetime use of menopausal hormone therapy (MHT) was identified using both self-reported information from the questionnaires sent out before 2004 and the drug reimbursement database since January 1, 2004. Education level, breastfeeding, age at menopause, age at menarche, parity and age at first full-term pregnancy, current level of physical activity, familial history of breast cancer, and lifetime use of oral contraceptives were generated from the biennial self-administered questionnaires sent before the study baseline. Body mass index (BMI), smoking status, lifetime personal history of benign breast disease, alcohol intake, self-report of a mammogram performed during the previous follow-up cycle, and lifetime histories of comorbidities that might be treated with glucocorticoids (rheumatism, arthrosis, and arthritis, spondyloarthritis, polyarthritis, asthma, chronic bronchitis, hay fever, or chronic inflammatory bowel diseases) originated from the biennial self-administered questionnaire sent before the study baseline with subsequent updates until 2011.

**Table 1:** Characteristics of participants, overall and according to glucocorticoid exposure at the end of follow-up

Characteristics at the end of follow-up^1^	All women (*n* = 62,512)	Exposure at the end of follow-up	
		Never/occasional users (*n* = 45,138)	Recurrent users (*n* = 17,374)
**Sociodemographic factors**			
**Age (years), mean (SD)**	72.1 (6.7)	71.8 (6.4)	72.8 (6.3)
**Educational level, N (%)**			
< High school	6516 (11)	4536 (10)	1980 (11)
From high school to 4 years higher education	45,138 (72)	32,771 (73)	12,367 (71)
At least 5 years higher education	10,858 (17)	7831 (17)	3027 (18)
**Lifestyle and reproductive factors**			
**BMI (kg/m**^**2**^**), N (%)**			
< 18.5	2626 (4)	1978 (4)	648 (4)
≥ 18.5 to < 23	25,409 (41)	19,037 (42)	6372 (37)
≥ 23 to < 25	13,197 (21)	9499 (21)	3698 (21)
≥ 25 to < 30	16,139 (26)	11,207 (25)	4932 (28)
≥ 30	5141 (8)	3417 (8)	1724 (10)
**Physical activity (Met-h/week), N (%)**			
≤ 34.8	15,649 (25)	11,115 (25)	4534 (26)
> 34.8 to ≤ 57.6	15,680 (25)	11,329 (25)	4351 (25)
> 57.6 to ≤ 88.8	15,567 (25)	11,306 (25)	4261 (25)
> 88.8	15,616 (25)	11,388 (25)	4228 (24)
**Smoking status, N (%)**			
Never smoker	33,281 (53)	24,229 (54)	9052 (52)
Current smoker	4741 (8)	3307 (7)	1434 (8)
Past smoker	24,490 (39)	17,602 (39)	6888 (40)
**Alcohol intake (g/day), N (%)**			
Abstainer	7832 (13)	5694 (13)	2138 (13)
≤ 5	16,796 (27)	12,198 (27)	4598 (26)
> 5 to ≤ 10	9357 (15)	6863 (15)	2494 (14)
> 10 to ≤ 20	12,053 (19)	8700 (19)	3353 (19)
> 20	12,522 (20)	8957 (20)	3565 (21)
Missing	3952 (6)	2726 (6)	1226 (7)
**Breastfeeding, N (%)**			
Never	23,425 (37)	16,829 (37)	6596 (38)
Ever	34,174 (55)	24,941 (55)	9233 (53)
Missing	4913 (8)	3368 (8)	1545 (9)
**Age at menopause (years), mean (SD)**	50.5 (3.7)	50.6 (3.7)	50.3 (3.9)
**Age at menarche (years), N (%)**			
< 13	28,078 (45)	20,054 (44)	8024 (46)
≥ 13	34,434 (55)	25,084 (56)	9350 (54)
**Parity and age at first full-term pregnancy, N (%)**			
Nulliparous	7282 (12)	5346 (12)	1936 (11)
First child before age 30 years, one or two children	31,393 (50)	22,434 (50)	8959 (51)
First child before age 30 years, three or more children	17,373 (28)	12,592 (28)	4781 (28)
First child after age 30 years	6464 (10)	4766 (11)	1698 (10)
**Lifetime oral contraceptive use, N (%)**	38,570 (62)	27,719 (61)	10,851 (62)
**Lifetime MHT use, N (%)**	45,239 (72)	31,843 (71)	13,396 (77)
**Number of medical consultations/visits during the preceding 6 months, N (%)**			
0	3277 (5)	2865 (6)	412 (2)
1 to 3	25,413 (41)	20,289 (45)	5124 (30)
≥ 4	33,491 (54)	21,667 (48)	11,824 (68)
Missing	331 (1)	317 (1)	14 (0)
**Self-report of a mammogram performed during the previous follow-up cycle, N (%)**	51,097 (82)	36,800 (82)	14,297 (82)
**Lifetime personal history of benign breast disease, N (%)**	23,268 (37)	16,435 (36)	6833 (39)
**History of breast cancer in first-degree relatives, N (%)**	7139 (11)	5196 (12)	1943 (11)
**Comorbidities, N (%)**			
**Lifetime history of arthrosis**	21,475 (34)	14,152 (31)	7323 (42)
**Lifetime history of rheumatisms**	6111 (10)	4504 (10)	1607 (9)
**Lifetime history of arthritis**	888 (1)	591 (1)	297 (2)
**Lifetime history of polyarthritis**	2375 (4)	1180 (3)	1195 (7)
**Lifetime history of spondyloarthritis**	236 (1)	123 (0)	113 (1)
**Lifetime history of asthma**	6043 (10)	3484 (8)	2559 (15)
**Lifetime history of chronic bronchitis**	7102 (11)	4278 (9)	2824 (16)
**Lifetime history of hay fever**	12,633 (20)	8629 (19)	4004 (23)
**Lifetime history of chronic inflammatory bowel diseases**	2284 (4)	1576 (3)	708 (4)
**Recurrent use of other drugs**^**2**^**, N (%)**			
**Nonsteroidal anti-inflammatory drugs**	38,493 (62)	14,238 (47)	24,255 (75)
**Immunosuppressants**	895 (1)	171 (0)	724 (4)
**Paracetamol**	38,392 (61)	24,725 (55)	13,667 (79)
**Proton pump inhibitors**	30,063 (48)	18,011 (40)	12,052 (69)
**Anti-arthritics**	26,400 (42)	17,095 (38)	9305 (54)

*Abbreviations*: *BMI* body mass index, *MHT* menopausal hormone therapy, *Met-h* metabolic equivalent task-hour, *SD* standard deviation

^1^Except for years of schooling, physical activity level, age at menarche, parity and age at first birth, lifetime use of oral contraceptives, history of breast cancer in first degree relatives and age at menopause which were assessed before the start of follow-up

^2^Recurrent use was defined as at least two reimbursements of the drug of interest during any previous 3-month period since January 1, 2004

### Statistical analysis

We used multivariable Cox regression models with age as the time scale and stratified by birth cohort (in 5-year categories) to estimate hazard ratios (HRs) and 95% confidence intervals (CIs) for the association of recurrent glucocorticoid use (versus occasional or never use) with breast cancer risk, overall and by breast cancer subtype. We considered exposure, other factors issued from the reimbursement database, and covariates updated during follow-up as time-varying parameters. Thus, for a given drug, participants contributed follow-up as unexposed until purchasing the drug for the second time in a 3-month period. The cumulative number of reimbursements, age at first use, and time since the first/last use were also updated during follow-up.

We systematically included in the multivariable models the following covariates: educational level, recent mammogram, and established risk factors for breast cancer (BMI, physical activity level, lifetime personal history of benign breast disease, family history of breast cancer, age at menarche, age at menopause, parity and age first full-term pregnancy, lifetime use of oral contraceptives, lifetime use of MHT, and alcohol consumption). The number of consultations with the doctor during the preceding 6 months and recurrent use of proton pump inhibitors modified the HR of certain breast cancer subtypes associated with exposure to glucocorticoids by at least 0.05 point and were therefore included in the final multivariable models. None of the other factors tested as potential confounding factors (breastfeeding, smoking status, lifetime histories of comorbidities that might be treated with glucocorticoids, or drugs likely to be used by glucocorticoid users) was included in the final models. The categories used are displayed in Table 1. For these covariates, when missing values represented < 5%, they were replaced either with the previous non-missing questionnaire value where appropriate or with the mode or the median values observed among the subjects with complete data. Only alcohol consumption and breastfeeding had ≥ 5% of missing values, which was accommodated by using a “missing” category in our models. A complete case analysis was also conducted (not shown because the results were similar).

We lagged by 6 months all variables coming from the reimbursement database (exposure, use of MHT, use of other drugs, and number of consultations) to allow a period of latency and to minimize reverse causation bias due to any early breast cancer symptoms [16]. The results were similar when exposure and other variables were lagged or not (data not shown).

We evaluated the effect modification by age, BMI, MHT use, comorbidities, and other drugs (all considered as time-varying parameters) by including cross-product interaction terms in the Cox models.

We performed a competing risk analysis using the cause-specific hazards approach to study the heterogeneity by tumour subtype/stage/grade [17, 18]. We censored women who developed the competing breast cancer subtypes at the time of occurrence and excluded cases with missing information on a given tumour characteristic from the corresponding analyses.

We conducted a sensitivity analysis in which women with no reimbursement of glucocorticoids served as the reference category (rather than women with either no or occasional reimbursements). Then, we repeated the analyses with exposed women defined as women with at least three reimbursements (women with less than three reimbursements served as the reference category). We also used a 2-year exposure lag instead of the 6-month lag. To assess the potential impact of surveillance bias, we restricted our analyses (i) to women who self-reported having had a mammogram performed during the previous follow-up cycle and (ii) to women with at least one medical consultation during the preceding 6 months.

All tests of statistical significance were two-sided, and significance was set at .05. We performed all analyses using the SAS software, version 9.4 (SAS Institute Inc., Cary, NC).

## Results

During a median follow-up time of 9 years, 2864 breast cancer cases were diagnosed (335 in situ, 2353 invasive, and 176 of unknown invasiveness status) among the 62,512 participants. Among the invasive cases, 2260 had information on ER status (1952 ER-positive and 308 ER-negative), 2206 on PR status (1459 PR-positive and 747 PR-negative), 2029 on HER2 status (244 HER2-positive and 1785 HER2-negative), 2326 on histological subtype (1741 ductal, 405 lobular, and 180 others), 2236 on grade (650 grade 1, 1183 grade 2, and 403 grade 3), and 2273 on stage (1501 stage 1, 610 stage 2, 126 stage 3, and 36 stage 4). Among the 62,512 participants, 30% had never been exposed, 42% had been occasionally exposed, and 28% had been recurrently exposed to systemic glucocorticoids during follow-up. Among the recurrent users, the most frequently reimbursed glucocorticoids were prednisone (39% of the total number of glucocorticoid reimbursements), prednisolone (30%), betamethasone (13%), and cortivazol (10%). Less than 8% of reimbursements were for dexamethasone, methylprednisolone, hydrocortisone, or triamcinolone.

Compared to never/occasional users, recurrent glucocorticoid users were older, had a higher BMI, had a more frequent medical follow-up, were more likely to have ever used MHT, and had more frequent histories of arthrosis, asthma, chronic bronchitis, or hay fever as well as recurrent exposure to nonsteroidal anti-inflammatory drugs, paracetamol, proton pump inhibitors, and anti-arthritics (Table 1).

**Fig. 1:**
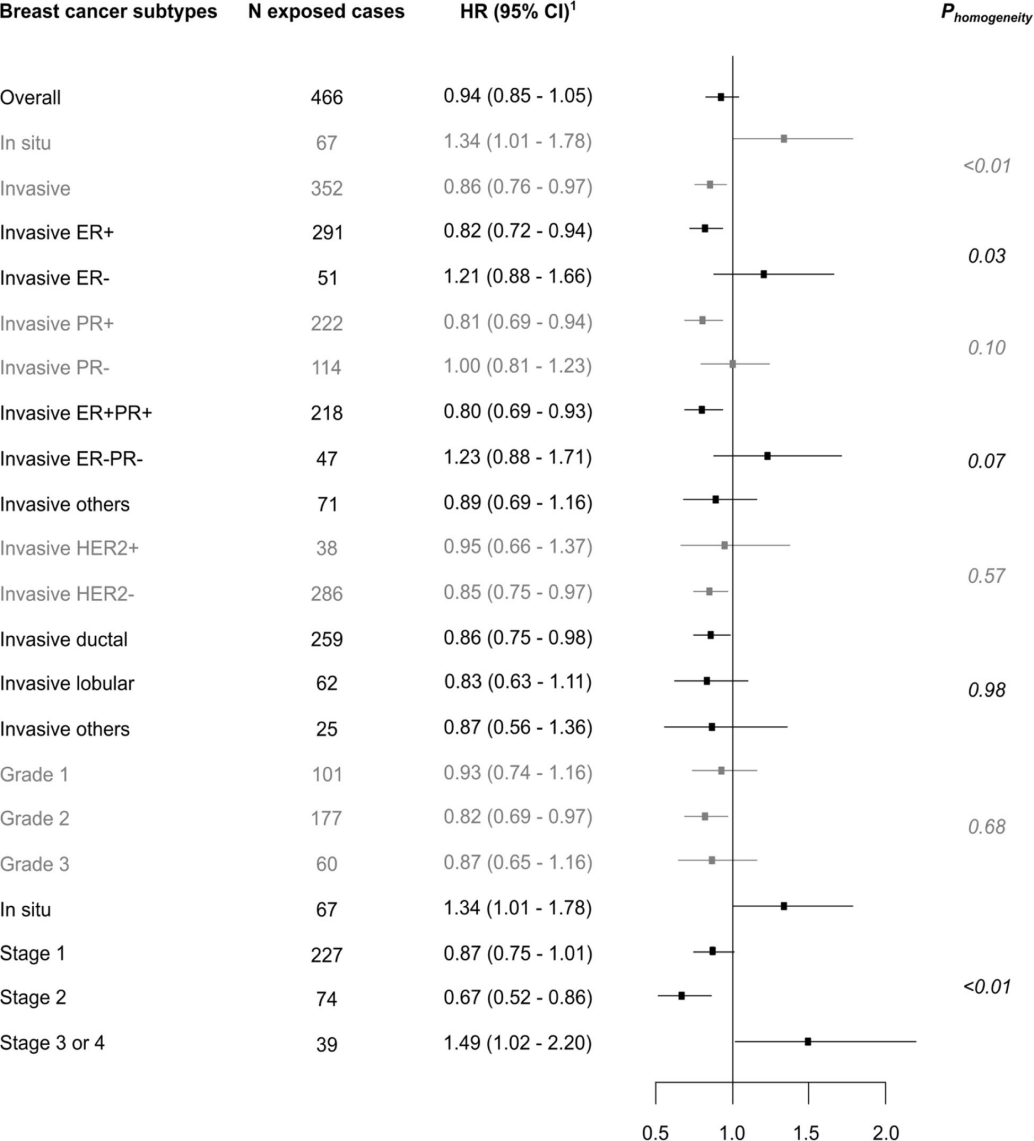
Glucocorticoid recurrent use and risk of different subtypes of breast cancer. Associations of glucocorticoid recurrent use with breast cancer risk, compared to never/occasional use, overall and breast cancer subtype (E3N Cohort; 2004 to 2014; *n* = 62,512). CI, confidence interval; HR, hazard ratio, ER, oestrogen receptor; PR, progesterone receptor; HER2, human epidermal growth factor receptor 2. ^1^HR adjusted for age (time scale), years of schooling (baseline), alcohol intake (time-varying), body mass index (time-varying), physical activity level (baseline), age at menarche (baseline), parity and age at first birth (baseline), lifetime use of oral contraceptives (baseline), age at menopause (baseline), history of breast cancer in first degree relatives (baseline), personal history of benign breast disease (time-varying), lifetime use of menopausal hormone therapy (time-varying), self-report of a mammogram performed during the previous follow-up cycle (time-varying), number of medical consultations/visits during the preceding 6 months (time-varying), and recurrent use of proton pump inhibitors (time-varying). Categories used are those displayed in Table 1.

The age-adjusted HR of breast cancer associated with having recurrently been exposed to glucocorticoids, compared with having never/occasionally been exposed, was 0.98 (95% CI 0.89–1.09). The multivariable HR was 0.94 (95% CI 0.85–1.05) (Fig. 1). Significant heterogeneity was found according to the invasiveness status (*P*_*homogeneity*_ < 0.01), with a positive association of glucocorticoid use with the risk of in situ breast cancer [HR = 1.34 (1.01–1.78)] and an inverse association with the risk of invasive breast cancer [HR = 0.86 (0.76–0.97)].

Regarding the risk of in situ breast cancer, no statistically significant heterogeneity/trend according to the type of glucocorticoid, route of administration, time since the first/last use, age at first use, or cumulative number of reimbursements was found (*P*_*trend/homogeneity*_ *≥ 0.11*, Additional file 1: Table S1).

Regarding the risk of invasive breast cancer, statistically significant heterogeneities by tumour stage (*P*_*homogeneity*_ < 0.01) and ER status (*P*_*homogeneity*_ = 0.03) were found (Fig. 1). Compared with never/occasional use, recurrent use of glucocorticoids was associated with lower risks of stage 1 and stage 2 breast cancer [HR_*stage1*_ = 0.87 (0.75–1.01); HR_*stage2*_ = 0.67 (0.52–0.86)] and a higher risk of stage 3/4 breast cancer [HR_stage3 or 4_ = 1.49 (1.02–2.20)]. The lower risk of invasive breast cancer was only found for ER+ breast cancer [HR_*ER+*_ = 0.82 (0.72–0.94); HR_*ER−*_ = 1.21 (0.88–1.66)]. The breast cancer-glucocorticoid association did not differ by other breast cancer characteristics (grade, HER2 status, PR status, histological type). No statistically significant heterogeneity/trend of invasive breast cancer risk according to the type of glucocorticoid, route of administration, time since first/last use, or age at first use was found (*P*_*trend/homogeneity*_ ≥ 0.29, Table 2). However, analyses according to the cumulative number of reimbursements yielded a statistically significant trend suggesting a lower risk of invasive breast cancer in the highest categories of reimbursements [>15 reimbursements: HR = 0.53 (0.34–0.81); *P*_*trend*_ = 0.02].

**Table 2:** Associations of glucocorticoid recurrent use with invasive breast cancer risk, according to the characteristics of use.

Characteristics of exposure	No. of cases	HR^1^ (95% CI)
Route of administration^2^		
Oral	198	0.82 (0.70–0.95)
Parenteral	130	0.90 (0.75–1.08)
*P*_*homogeneity*_		*0.40*
Type of glucocorticoid^3^		
Betamethasone	56	0.88 (0.66–1.14)
Prednisolone	128	0.90 (0.75–1.08)
Prednisone	47	0.80 (0.59–1.07)
Cortivazol	57	0.94 (0.72–1.23)
Others^4^	10	0.47 (0.25–0.87)
*P*_*homogeneity*_		*0.98*
Cumulative number of reimbursements		
Occasional/never use	2001	1.00 (ref)
≤ 5	179	0.88 (0.75–1.03)
> 5 to ≤ 10	84	0.84 (0.67–1.05)
> 10 to ≤ 15	18	0.68 (0.42–1.08)
> 15	21	0.53 (0.34–0.81)
Unknown	50	1.16 (0.87–1.53)
*P*_*trend*_^5^		*0.02*
Time since the first use (years)		
Occasional/never use	2001	1.00 (ref)
≤ 2	112	0.88 (0.73–1.07)
> 2 to ≤ 4	76	0.77 (0.61–0.98)
> 4 to ≤ 6	61	0.86 (0.66–1.11)
> 6	69	0.92 (0.72–1.18)
Unknown	34	0.86 (0.61–1.21)
*P*_*trend*_^5^		*0.82*
Time since the last use (years)		
Occasional/never use	2001	1.00 (ref)
≤ 1	255	0.84 (0.74–0.97)
> 1 to ≤ 2	44	0.96 (0.71–1.29)
> 2 to ≤ 3	25	0.94 (0.63–1.40)
> 3 to ≤ 4	12	0.76 (0.43–1.35)
> 4	16	0.78 (0.48–1.28)
*P*_*trend*_^5^		*0.81*
Age at first use (years)		
Occasional/never use	2001	1.00 (ref)
≤ 60	79	0.96 (0.76–1.22)
> 60 to ≤ 70	161	0.81 (0.69–0.96)
> 70	67	0.73 (0.57–0.95)
Unknown	45	1.09 (0.81–1.47)
*P*_*trend*_^5^		*0.29*

*Abbreviations*: *CI* confidence interval, *HR* hazard ratio

^1^HR adjusted for age (time scale), years of schooling (baseline), alcohol intake (time-varying), body mass index (time-varying), physical activity level (baseline), age at menarche (baseline), parity and age at first birth (baseline), lifetime use of oral contraceptives (baseline), age at menopause (baseline), history of breast cancer in first-degree relatives (baseline), personal history of benign breast disease (time-varying), lifetime use of menopausal hormone therapy (time-varying), self-report of a mammogram performed during the previous follow-up cycle (time-varying), number of medical consultations/visits during the preceding 6 months (time-varying), and recurrent use of proton pump inhibitors (time-varying). Categories used are those displayed in Table 1. HRs were obtained from separate models including one characteristic of exposure at a time

^2^Variables corresponding to the recurrent use (versus never/occasional use) of oral/parenteral glucocorticoids were introduced simultaneously in the model. A woman who had taken oral and parenteral glucocorticoids would contribute to both categories

^3^Variables corresponding to the recurrent use (versus never/occasional use) of each type of glucocorticoid displayed in the table were introduced simultaneously in the model. A woman who had taken different types of glucocorticoids would contribute to several categories

^4^Other molecules include dexamethasone, methylprednisolone, triamcinolone, and hydrocortisone

^5^Tests for linear trends were performed among recurrently exposed women with known characteristics of exposure, using an ordinal variable across categories. The corresponding variable was introduced in the models as continuous

No effect modification was found by current age, BMI, ever use of MHT, selected comorbidities, and use of selected drugs (*P*_*interaction*_ ≥ 0.24, Additional file 1: Figure S1).

Table S2 (Additional file 1) shows that, when compared with never users, only recurrent users (not occasional users) of glucocorticoids were at higher risk of in situ or stage 3/4 breast cancer, and only recurrent users were at lower risk of stage 1 or 2 breast cancer. When we changed the definition of exposure to at least three reimbursements, the results remained virtually unchanged (data not shown). When we applied a lag-time of 2 years (Additional file 1: Figure S2), or restricted our analyses to women with a recent mammogram (Additional file 1: Figure S3) or to women with at least one medical consultation during the preceding 6 months (Additional file 1: Table S3), the direction of all of the main findings was the same as the primary analysis. However, some associations did not reach statistical significance due to the more limited available sample size for these sub-analyses.

## Discussion

### Summary of findings

In this large cohort of postmenopausal women, glucocorticoid exposure was associated with a lower risk of invasive breast cancer that was restricted to ER+ and to stage 1 or 2 tumours and that was more marked in the highest categories of the cumulative number of reimbursements. Conversely, glucocorticoid use was associated with higher risks of in situ and stage 3/4 breast cancers.

### Comparison with previous studies

The potential impact of glucocorticoids on breast cancer incidence has been rarely evaluated in epidemiological studies. The only epidemiological study published to date to specifically evaluate glucocorticoid-breast cancer associations reported a null association between glucocorticoid use and invasive breast cancer risk [systemic glucocorticoids: odds ratio = 1.00 (0.96–1.10), n exposed cases = 908] [13]. The associations did not differ by intensity or duration of use. The authors were not able to stratify their analyses by molecular status and tumour stage of breast cancer, which makes any comparison with our results difficult.

However, our results, suggesting a lower risk of invasive breast cancer among glucocorticoids users, especially for ER+ tumours, are supported by experimental models showing that the expression of the glucocorticoid receptor was correlated with improved breast cancer prognosis especially for ER+ tumours and that activation of the glucocorticoid receptor may reduce oestrogen-induced cell proliferation in ER+ breast cancer [19, 20]. Indeed, glucocorticoids were shown to have preventive breast cancer effects by stimulating the expression of sulfotransferase SULT1E1 (which plays a role in deactivating oestrogens) [4].

Other mechanisms which could lead to a decreased breast cancer risk include the effects of glucocorticoids on angiogenesis [21], or the inhibition of inflammatory and growth factors [22, 23]. We found no study on the impact of glucocorticoids on in situ breast tumours, but our finding indicating an increased risk of in situ breast cancer and a decreased risk of stage 1 and 2 breast cancer with glucocorticoid exposure could be explained by the fact that previous or current exposure to glucocorticoids might hamper breast tumour progression from in situ to invasive phenotype and therefore keep the cancer cells located in situ longer.

The higher risk of stage 3/4 breast cancers with glucocorticoid exposure is in line with other previous experiments showing that the glucocorticoid receptor was overexpressed in metastatic breast cancer and might be a strong inducer of epithelial-to-mesenchymal transition [24]. A recent experiment in mice with breast cancer suggested that glucocorticoid use might activate glucocorticoid receptors at distant metastatic sites and increase cancer cell growth, promote breast cancer metastasis, and reduce survival [25]. In addition, glucocorticoids have been hypothesized to promote breast cancer progression and metastasis through the activation of the TEA domain transcription factor 4 [26]. Studies conducted in the 1960s among breast cancer patients reported that the use of adrenal steroids (including glucocorticoids), compared to non-use, increased the risk of metastasis [27].

### Interpretation of results and implications

Although in line with some experimental studies, the mechanistic interpretation of our epidemiological findings is unclear. In particular, the potential dual effects of glucocorticoids on breast cancer development according to tumour stage have never been evaluated in previous experimental studies. The mechanisms underlying the potential impact of glucocorticoids according to tumour stage should be explored in experimental studies, and our results replicated in larger epidemiological studies with information on breast cancer subtypes/stages. If our results are confirmed, this will have to be taken into account in the risk-benefit assessment of glucocorticoid prescriptions made by clinicians and/or in recommending mammographic control before initiating glucocorticoid treatment. It is important to mention that glucocorticoids are occasionally used among breast cancer patients to reduce chemotherapy-induced side effects such as nausea or lack of appetite. Thus, further studies should also consider the potential impact on breast cancer metastasis of such use.

### Strengths and limitations

The main strengths of this study included its prospective design and the use of information from a drug reimbursement database to identify glucocorticoid exposure, which avoids differential recall bias between cases and non-cases. In addition, the exhaustive data on glucocorticoid reimbursements allowed us to consider precise information on exposure (including the number of reimbursements and timing of use). However, we lacked data regarding the compliance/adherence to the dispensed treatment, but defining users as women with at least two reimbursements during a 3-month period makes it likely that they took the drug. Since data on glucocorticoid reimbursements were combined with self-reported data on lifestyle, reproductive, and medical factors, we were able to take into account potential confounders. Surveillance bias could arise due to more frequent medical consultations or more frequent mammographic screening for women prescribed glucocorticoids and thus increased likelihood of being diagnosed with breast cancer (in particular, in situ breast cancer, which is often found during a mammogram done as part of breast cancer screening). We addressed that bias by adjusting our models for a recent mammogram and the number of recent medical consultations. As sensitivity analyses, we also restricted our analyses to women with a recent mammogram or to women with at least one medical consultation during the preceding 6 months. These additional restrictions were not in favour of surveillance bias since the results remained unchanged. However, residual confounding by medical surveillance issues cannot be excluded. We were also able to stratify the analyses according to breast cancer subtype and to examine interactions of glucocorticoid use with breast cancer risk factors, comorbidities, and other drugs. However, the power to detect differences was limited because of the low number of cases in some categories and the number of advanced-stage cases was too small to separate stage 3 and stage 4 breast cancers. It is also possible that our results are due to chance, and they must be interpreted carefully. In addition, while some autoimmune diseases were associated with breast cancer risk [28], we were not able to adjust or stratify on several comorbidities of interest such as adrenal insufficiency or some autoimmune diseases (e.g. systemic lupus erythematosus, pernicious anaemia, or psoriasis). Nevertheless, the prevalence of these comorbidities is low, and because they are not established breast cancer risk factors, we do not expect that they would confound the glucocorticoid-breast cancer associations.

## Conclusions

In this large cohort of postmenopausal women, glucocorticoid exposure was associated with a lower risk of invasive breast cancer that was restricted to ER+ and to stage 1 or 2 tumours and with a higher risk of in situ and stage 3/4 breast cancers.

These results are novel and suggest that tumour molecular subtype and stage are important to consider when evaluating the associations between glucocorticoid use and breast cancer risk. In-depth experimental and epidemiological researches are needed to better understand the potential impact of glucocorticoids on breast cancer risk.

## Supplementary Information

**Additional file 1: **Use of systemic glucocorticoids and risk of breast cancer in a prospective cohort of postmenopausal women: Supplementary Tables S1-S3 and Supplementary Figures S1-S3. **Table S1.** Associations of glucocorticoid recurrent use with *in situ* breast cancer risk, according to characteristics of use. **Table S2.** Associations of glucocorticoid recurrent and occasional use with breast cancer risk, compared to never use. **Table S3.** Associations of glucocorticoid recurrent use with breast cancer risk, compared to never/occasional use, overall and by breast cancer subtype, among women with at least one medical consultation during the preceding 6 months. **Figure S1.** Associations of glucocorticoid recurrent use with invasive breast cancer risk, compared to never/occasional use, in strata of selected factors, comorbidities and recurrent use of other drugs. **Figure S2.** Associations of glucocorticoid recurrent use with breast cancer risk, compared to never/occasional use, overall and by breast cancer subtype, with exposure and other covariates coming from the reimbursement database lagged by 2 years. **Figure S3.** Associations of glucocorticoid recurrent use with breast cancer risk, compared to never/occasional use, overall and by breast cancer subtype, among women with a recent mammogram.

## Data Availability

The data and computing code required to replicate the results reported in this paper are available upon duly motivated request by contacting Dr. Agnès Fournier.

## Abbreviations

BMI: Body mass index
CI: Confidence interval
ER: Oestrogen receptor
HER2: Human epidermal growth factor receptor 2
HR: Hazard ratio
MHT: Menopausal hormone therapy
PR: Progesterone receptor
