# Supplementary material for: Use of systemic glucocorticoids and risk of breast cancer in a prospective cohort of postmenopausal women
Source: BMC Med. 2021 Aug 2;19:186. doi: 10.1186/s12916-021-02004-6 (PMC8330083; doi:10.1186/s12916-021-02004-6)
Supplement: Supplementary file 1 — Additional file 1: Use of systemic glucocorticoids and risk of breast cancer in a prospective cohort of postmenopausal women: Supplementary Tables S1-S3 and Supplementary Figures S1-S3. Table S1. Associations of glucocorticoid recurrent use with in situ breast cancer risk, according to characteristics of use. Table S2. Associations of glucocorticoid recurrent and occasional use with breast cancer risk, compared to never use. Table S3. Associations of glucocorticoid recurrent use with breast cancer risk, compared to never/occasional use, overall and by breast cancer subtype, among women with at least one medical consultation during the preceding 6 months. Figure S1. Associations of glucocorticoid recurrent use with invasive breast cancer risk, compared to never/occasional use, in strata of selected factors, comorbidities and recurrent use of other drugs. Figure S2. Associations of glucocorticoid recurrent use with breast cancer risk, compared to never/occasional use, overall and by breast cancer subtype, with exposure and other covariates coming from the reimbursement database lagged by 2 years. Figure S3. Associations of glucocorticoid recurrent use with breast cancer risk, compared to never/occasional use, overall and by breast cancer subtype, among women with a recent mammogram. [file 12916_2021_2004_MOESM1_ESM.docx]

**Additional file 1.**

**Use of systemic glucocorticoids and risk of breast cancer in a prospective cohort of postmenopausal women: Supplementary Tables S1-S3 and Supplementary Figures S1-S3.**

**Table S1.** Associations of glucocorticoid recurrent use with *in situ* breast cancer risk, according to characteristics of use (E3N Cohort; 2004 to 2014; n= 62,512).

| **Characteristics of exposure** | **N cases** | **HR**^1^ **(95% CI)** |
| --- | --- | --- |
| **Route of administration^2^** |  |  |
| Oral | 46 | 1.50 (1.09 – 2.08) |
| Parenteral | 19 | 0.93 (0.58 – 1.50) |
| *P_homogeneity_* |  | *0.86* |
| **Type of glucocorticoid^3^** |  |  |
| [Betamethasone](https://www.whocc.no/atc_ddd_index/?code=H02AB01&showdescription=yes) | 6 | 0.61 (0.27 – 1.37) |
| [Prednisolone](https://www.whocc.no/atc_ddd_index/?code=H02AB06&showdescription=yes) | 25 | 1.22 (0.80 – 1.86) |
| [Prednisone](https://www.whocc.no/atc_ddd_index/?code=H02AB07&showdescription=yes) | 14 | 1.63 (0.94 – 2.85) |
| [Cortivazol](https://www.whocc.no/atc_ddd_index/?code=H02AB17&showdescription=yes) | 9 | 1.07 (0.55 – 2.11) |
| Other glucocorticoids^4^ | 8 | 2.48 (1.20 – 5.11) |
| *P_homogeneity_* |  | *0.11* |
| **Cumulative number of reimbursements** | | |
| Occasional/never use | 268 | 1.00 (ref) |
| ≤ 5 | 31 | 1.24 (0.85 – 1.92) |
| > 5 to ≤ 10 | 17 | 1.43 (0.86 – 2.37) |
| > 10 | 12 | 1.46 (0.80 – 2.65) |
| Unknown | 7 | 1.44 (0.68 – 3.06) |
| *P_trend_^5^* |  | *0.45* |
| **Time since first use (years)** |  |  |
| Occasional/never use | 268 | 1.00 (ref) |
| < 2 | 20 | 1.29 (0.82 – 2.06) |
| > 2 to ≤ 4 | 17 | 1.45 (0.88 – 2.40) |
| > 4 to ≤ 6 | 13 | 1.51 (0.85 – 2.68) |
| > 6 | 11 | 1.19 (0.63 – 2.23) |
| Unknown | 6 | 1.20 (0.53 – 2.72) |
| *P_trend_^5^* |  | *0.83* |
| **Time since last use (years)** |  |  |
| Occasional/never use | 268 | 1.00 (ref) |
| < 1 | 47 | 1.28 (0.92 – 1.77) |
| > 1 to ≤ 2 | 13 | 2.29 (1.30 – 4.03) |
| > 2 | 7 | 0.91 (0.43 – 1.94) |
| *P_trend_^5^* |  | *0.79* |
| **Age at first use (years)** |  |  |
| Occasional/never use | 268 | 1.00 (ref) |
| ≤ 60 | 12 | 0.90 (0.50 – 1.65) |
| > 60 to ≤ 70 | 34 | 1.37 (0.93 – 2.00) |
| > 70 | 11 | 1.48 (0.76 – 2.90) |
| Unknown | 10 | 2.25 (1.18 – 4.29) |
| *P_trend_^5^* |  | *0.66* |

Abbreviations: CI, confidence interval; HR, hazard ratio.

^1^ HR adjusted for age (time scale), years of schooling (baseline), alcohol intake (time-varying), body mass index (time-varying), physical activity level (baseline), age at menarche (baseline), parity and age at first birth (baseline), lifetime use of oral contraceptives (baseline), age at menopause (baseline), history of breast cancer in first degree relatives (baseline), personal history of benign breast disease (time-varying), lifetime use of menopausal hormone therapy (time-varying), self–report of a mammogram performed during the previous follow–up cycle (time-varying), number of medical consultations/visits during the preceding 6 months (time-varying), and recurrent use of proton pump inhibitors (time-varying). Categories used are those displayed in Table 1. HRs were obtained from separate models including one characteristic of exposure at a time.

^2^ Variables corresponding to recurrent use (versus never/occasional use) of oral / parenteral glucocorticoids were introduced simultaneously in the model. A woman who had taken oral and parenteral glucocorticoids would contribute to both categories.

^3^ Variables corresponding to recurrent use (versus never/occasional use) of each type of glucocorticoid displayed in the table were introduced simultaneously in the model. A woman who had taken different types of glucocorticoids would contribute to several categories.

^4^ Other molecules include dexamethasone, methylprednisolone, triamcinolone, and hydrocortisone.

^5^ Tests for linear trends were performed among recurrently exposed women with known characteristics of exposure, using an ordinal variable across categories. The corresponding variable was introduced in the models as continuous.

**Table S2.** Associations of glucocorticoid recurrent and occasional use with breast cancer risk, compared to never use (E3N Cohort; 2004 to 2014; n=62,512).

|  | **N cases** | **HR^1^ (95% CI)** |
| --- | --- | --- |
| **Overall breast cancer** | | |
| Never use of glucocorticoids | 1,443 | 1 (reference) |
| Occasional use of glucocorticoids | 955 | 0.95 (0.87 – 1.03) |
| Recurrent use of glucocorticoids | 466 | 0.91 (0.82 – 1.03) |
| ***In situ* breast cancer** |  |  |
| Never use of glucocorticoids | 167 | 1 (reference) |
| Occasional use of glucocorticoids | 101 | 0.92 (0.71 – 1.20) |
| Recurrent use of glucocorticoids | 67 | 1.28 (0.94 – 1.76) |
| **Invasive breast cancer** |  |  |
| Never use of glucocorticoids | 1,206 | 1 (reference) |
| Occasional use of glucocorticoids | 795 | 0.95 (0.86 – 1.04) |
| Recurrent use of glucocorticoids | 352 | 0.83 (0.73 – 0.95) |
| **Invasive ER+ breast cancer** |  |  |
| Never use of glucocorticoids | 995 | 1 (reference) |
| Occasional use of glucocorticoids | 666 | 0.93 (0.84 – 1.03) |
| Recurrent use of glucocorticoids | 291 | 0.79 (0.69 – 0.91) |
| **Invasive ER- breast cancer** |  |  |
| Never use of glucocorticoids | 162 | 1 (reference) |
| Occasional use of glucocorticoids | 95 | 1.02 (0.78 – 1.34) |
| Recurrent use of glucocorticoids | 51 | 1.22 (0.86 – 1.73) |
| **Invasive PR+ breast cancer** |  |  |
| Never use of glucocorticoids | 720 | 1 (reference) |
| Occasional use of glucocorticoids | 517 | 0.96 (0.85 – 1.09) |
| Recurrent use of glucocorticoids | 222 | 0.79 (0.67 – 0.93) |
| **Invasive PR- breast cancer** |  |  |
| Never use of glucocorticoids | 405 | 1 (reference) |
| Occasional use of glucocorticoids | 228 | 0.89 (0.75 – 1.05) |
| Recurrent use of glucocorticoids | 114 | 0.94 (0.75 – 1.18) |
| **Invasive HER2+ breast cancer** |  |  |
| Never use of glucocorticoids | 124 | 1 (reference) |
| Occasional use of glucocorticoids | 82 | 0.98 (0.73 – 1.32) |
| Recurrent use of glucocorticoids | 38 | 0.94 (0.64 – 1.40) |
| **Invasive HER2- breast cancer** |  |  |
| Never use of glucocorticoids | 854 | 1 (reference) |
| Occasional use of glucocorticoids | 645 | 0.98 (0.88 – 1.09) |
| Recurrent use of glucocorticoids | 286 | 0.84 (0.73 – 0.97) |
| **Grade 1 breast cancer** |  |  |
| Never use of glucocorticoids | 347 | 1 (reference) |
| Occasional use of glucocorticoids | 202 | 0.86 (0.71 – 1.03) |
| Recurrent use of glucocorticoids | 101 | 0.86 (0.67 – 1.09) |
| **Grade 2 breast cancer** |  |  |
| Never use of glucocorticoids | 564 | 1 (reference) |
| Occasional use of glucocorticoids | 442 | 1.10 (0.97 – 1.26) |
| Recurrent use of glucocorticoids | 177 | 0.87 (0.72 – 1.04) |
| **Grade 3 breast cancer** |  |  |
| Never use of glucocorticoids | 225 | 1 (reference) |
| Occasional use of glucocorticoids | 118 | 0.73 (0.58 – 0.93) |
| Recurrent use of glucocorticoids | 60 | 0.74 (0.55 – 1.01) |
| ***In situ* breast cancer** |  |  |
| Never use of glucocorticoids | 167 | 1 (reference) |
| Occasional use of glucocorticoids | 101 | 0.92 (0.71 – 1.20) |
| Recurrent use of glucocorticoids | 67 | 1.28 (0.94 – 1.76) |
| **Stage 1 breast cancer** |  |  |
| Never use of glucocorticoids | 772 | 1 (reference) |
| Occasional use of glucocorticoids | 502 | 0.92 (0.82 – 1.04) |
| Recurrent use of glucocorticoids | 227 | 0.83 (0.71 – 0.98) |
| **Stage 2 breast cancer** |  |  |
| Never use of glucocorticoids | 323 | 1 (reference) |
| Occasional use of glucocorticoids | 213 | 0.99 (0.82 – 1.19) |
| Recurrent use of glucocorticoids | 74 | 0.67 (0.51 – 0.87) |
| **Stage 3 or 4 breast cancer** | | |
| Never use of glucocorticoids | 71 | 1 (reference) |
| Occasional use of glucocorticoids | 52 | 1.03 (0.70 – 1.50) |
| Recurrent use of glucocorticoids | 39 | 1.52 (0.98 – 2.34) |

Abbreviations: CI, confidence interval; HR, hazard ratio.

^1^ HR adjusted for age (time scale), years of schooling (baseline), alcohol intake (time-varying), body mass index (time-varying), physical activity level (baseline), age at menarche (baseline), parity and age at first birth (baseline), lifetime use of oral contraceptives (baseline), age at menopause (baseline), history of breast cancer in first degree relatives (baseline), personal history of benign breast disease (time-varying), lifetime use of menopausal hormone therapy (time-varying), self-report of a mammogram performed during the previous follow-up cycle (time-varying), number of medical consultations/visits during the preceding 6 months (time-varying), and recurrent use of proton pump inhibitors (time-varying). Categories used are those displayed in Table 1.

**Table S3.** Associations of glucocorticoid recurrent use with breast cancer risk, compared to never/occasional use, overall and by breast cancer subtype, among women with at least one medical consultation during the preceding 6 months (E3N Cohort; 2004 to 2014; n=58,904).

| **Breast cancer subtype** | **N exposed cases** | **HR^1^ (95% CI)** | ***P_homogeneity_*** |
| --- | --- | --- | --- |
| Overall | 458 | 0.95 (0.85 – 1.06) |  |
|  |  |  |  |
| *In situ*  Invasive | 67  345 | 1.36 (1.02 – 1.82)  0.86 (0.76 – 0.97) | <0.01 |
|  |  |  |  |
| ER+  ER- | 286  50 | 0.83 (0.73 – 0.95)  1.21 (0.87 – 1.67) | 0.04 |
|  |  |  |  |
| *In situ*  Stage 1  Stage 2  Stage 3 or 4 | 67  224  72  37 | 1.36 (1.02 – 1.82)  0.88 (0.76 – 1.02)  0.67 (0.52 – 0.87)  1.43 (0.96 – 2.12) | <0.01 |

Abbreviations: CI, confidence interval; HR, hazard ratio.

^1^ HR adjusted for age (time scale), years of schooling (baseline), alcohol intake (time-varying), body mass index (time-varying), physical activity level (baseline), age at menarche (baseline), parity and age at first birth (baseline), lifetime use of oral contraceptives (baseline), age at menopause (baseline), history of breast cancer in first degree relatives (baseline), personal history of benign breast disease (time-varying), lifetime use of menopausal hormone therapy (time-varying), self-report of a mammogram performed during the previous follow-up cycle (time-varying), number of medical consultations/visits during the preceding 6 months (time-varying), and recurrent use of proton pump inhibitors (time-varying). Categories used are those displayed in Table 1.

**Figure S1.** Associations of glucocorticoid recurrent use with invasive breast cancer risk, compared to never/occasional use, in strata of selected factors, comorbidities and recurrent use^1^ of other drugs (E3N Cohort; 2004 to 2014; n= 62,512).


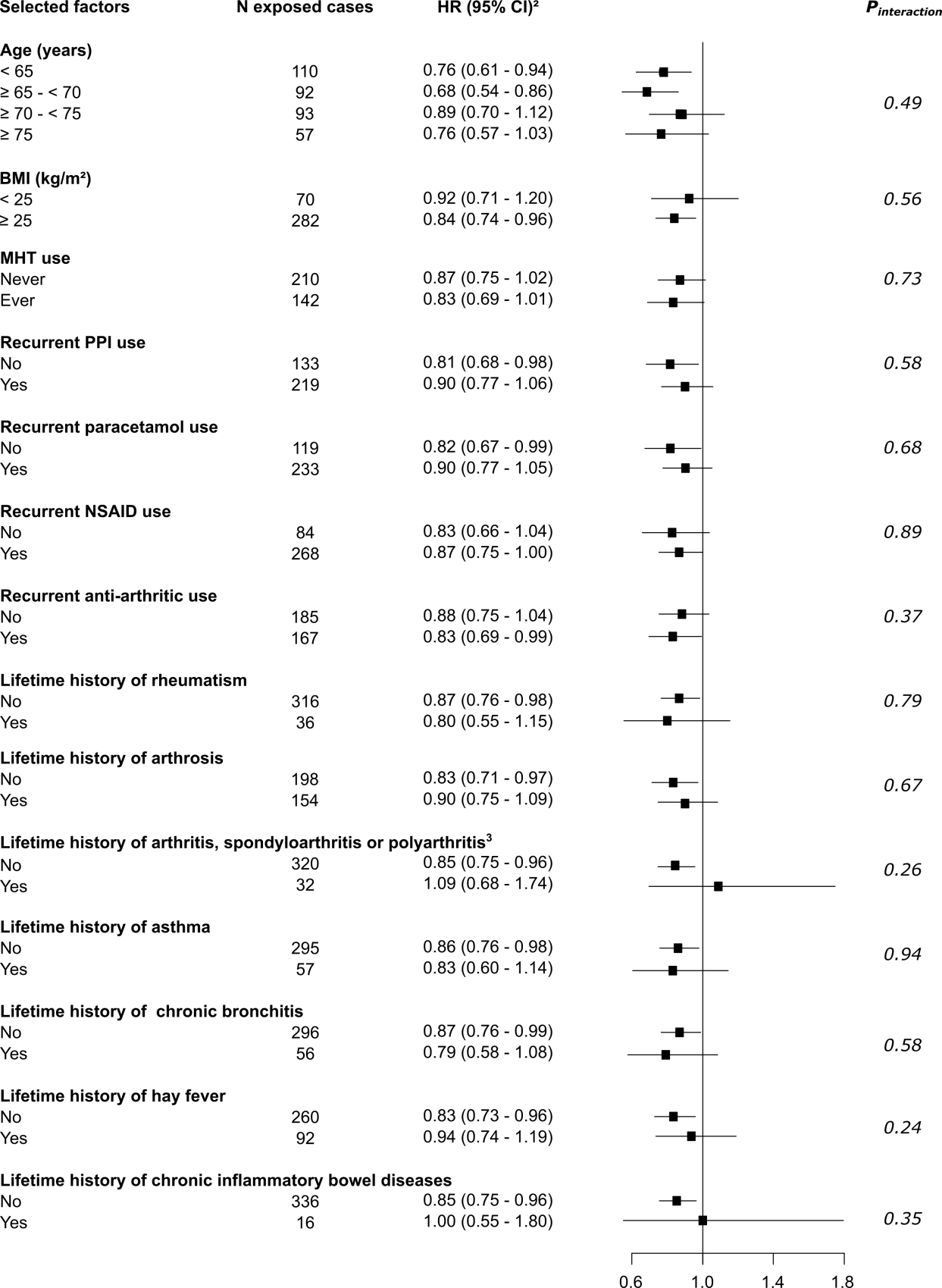


Abbreviations: BMI: body mass index, CI, confidence interval; HR, hazard ratio; MHT, menopausal hormone therapy; NSAID, nonsteroidal anti-inflammatory drug; PPI, proton pump inhibitor.

^1^ At least two reimbursements during any previous three-month period since January 1, 2004.

^2^ HR adjusted for age (time scale), years of schooling (baseline), alcohol intake (time-varying), body mass index (time-varying), physical activity level (baseline), age at menarche (baseline), parity and age at first birth (baseline), lifetime use of oral contraceptives (baseline), age at menopause (baseline), history of breast cancer in first degree relatives (baseline), personal history of benign breast disease (time-varying), lifetime use of menopausal hormone therapy (time-varying), self–report of a mammogram performed during the previous follow–up cycle (time-varying), number of medical consultations/visits during the preceding 6 months (time-varying), and recurrent use of proton pump inhibitors (time-varying). Categories used are those displayed in Table 1.

^3^ Associations were similar when comorbidities were ungrouped.

**Figure S2.** Associations of glucocorticoid recurrent use with breast cancer risk, compared to never/occasional use, overall and by breast cancer subtype, with exposure and other covariates coming from the reimbursement database lagged by 2 years (E3N Cohort; 2004 to 2014; n=62,512).


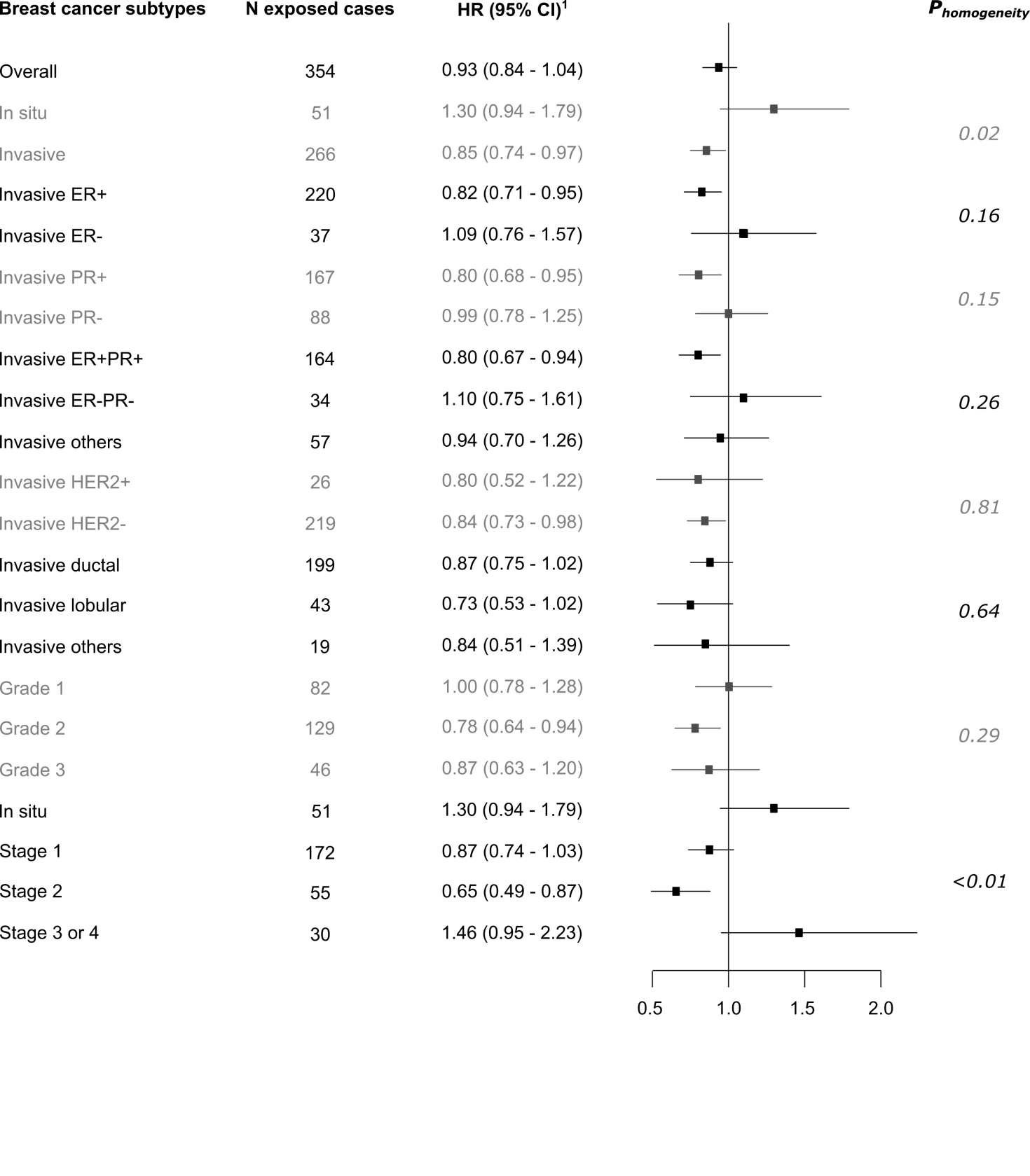


Abbreviations: CI, confidence interval; HR, hazard ratio; ER, estrogen receptor; PR, progesterone receptor; HER2, human epidermal growth factor receptor 2.

^1^HR adjusted for age (time scale), years of schooling (baseline), alcohol intake (time-varying), body mass index (time-varying), physical activity level (baseline), age at menarche (baseline), parity and age at first birth (baseline), lifetime use of oral contraceptives (baseline), age at menopause (baseline), history of breast cancer in first degree relatives (baseline), personal history of benign breast disease (time-varying), lifetime use of menopausal hormone therapy (time-varying), self-report of a mammogram performed during the previous follow-up cycle (time-varying), number of medical consultations/visits during the preceding 6 months (time-varying), and recurrent use of proton pump inhibitors (time-varying). Categories used are those displayed in Table 1.

**Figure S3.** Associations of glucocorticoid recurrent use with breast cancer risk, compared to never/occasional use, overall and by breast cancer subtype, among women with a recent mammogram (E3N Cohort; 2004 to 2014; n=51,097).


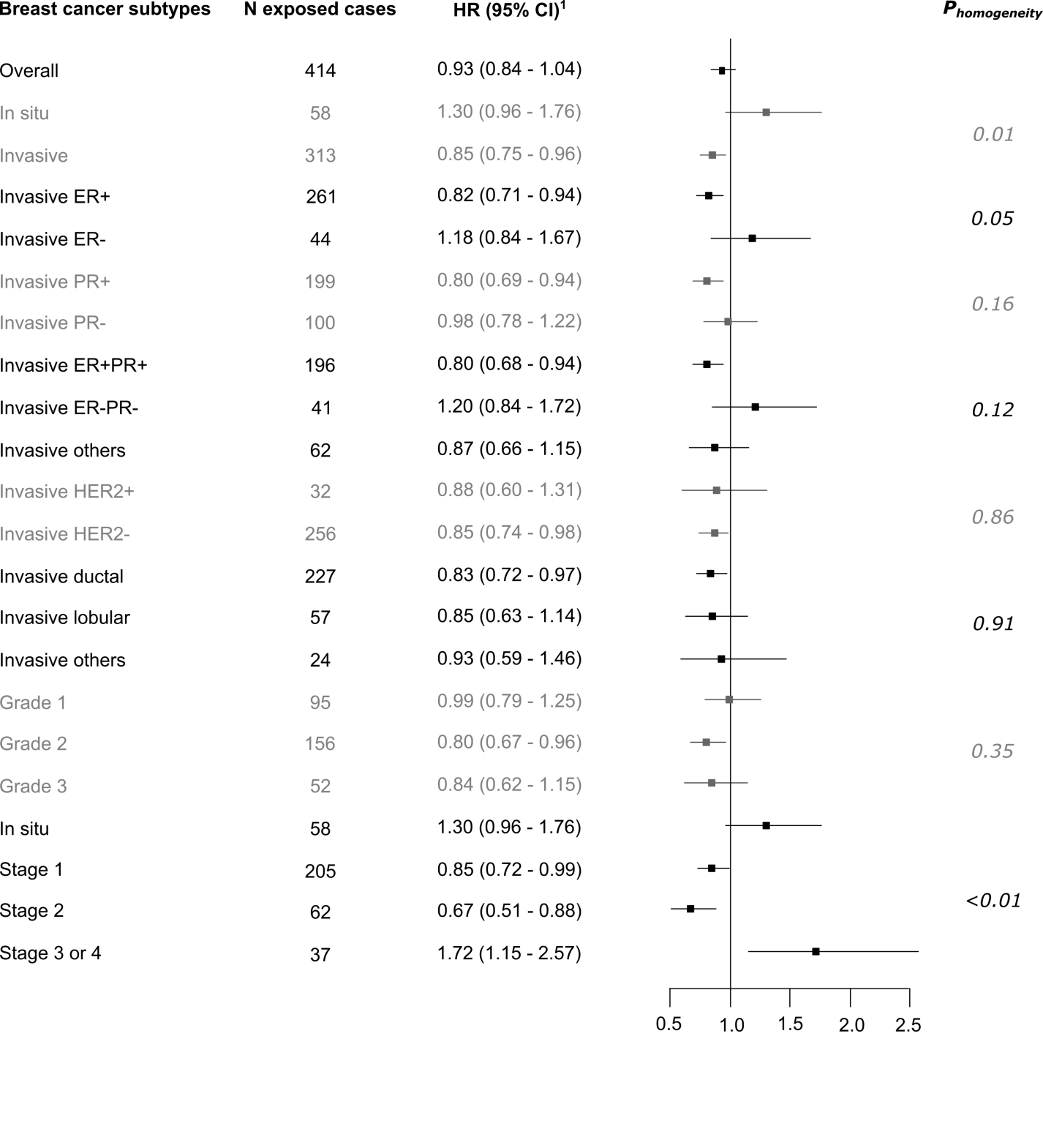


Abbreviations: CI, confidence interval; HR, hazard ratio; ER, estrogen receptor; PR, progesterone receptor; HER2, human epidermal growth factor receptor 2.

^1^HR adjusted for age (time scale), years of schooling (baseline), alcohol intake (time-varying), body mass index (time-varying), physical activity level (baseline), age at menarche (baseline), parity and age at first birth (baseline), lifetime use of oral contraceptives (baseline), age at menopause (baseline), history of breast cancer in first degree relatives (baseline), personal history of benign breast disease (time-varying), lifetime use of menopausal hormone therapy (time-varying), number of medical consultations/visits during the preceding 6 months (time-varying), and recurrent use of proton pump inhibitors (time-varying). Categories used are those displayed in Table 1.
